# Living With Symptom Experiences in Patients With Heart Failure: A Scoping Review

**DOI:** 10.7759/cureus.107667

**Published:** 2026-04-24

**Authors:** Tomohide Fukuda, Junichi Hosogaya, Yoshie Imai, Yoshie Murakami

**Affiliations:** 1 Faculty of Nursing, Musashino University, Tokyo, JPN; 2 Department of Nursing, Kawaguchi Cardiovascular and Respiratory Hospital, Saitama, JPN; 3 Faculty of Medicine, Tokushima University, Tokushima, JPN; 4 Faculty of Nursing and Medical Care, Keio University, Tokyo, JPN

**Keywords:** heart failure, patient reported experience, self-care, symptom experience, symptom recognition

## Abstract

Heart failure is a long-term progressive disease affecting millions of people worldwide, imposing physical, psychological, and social burdens on patients and their families. Symptom recognition, which refers to awareness and understanding of bodily changes, is a key aspect of self-care; however, how patients and their families live with symptoms and deal with them in their daily lives remains unclear. This review aimed to summarise the current knowledge on how patients with heart failure and their caregivers experience, notice, and manage symptoms in daily life, and to identify the knowledge gaps that make timely and appropriate care difficult.

This scoping review followed the frameworks of Arksey and O’Malley and the Preferred Reporting Items for Systematic Reviews and Meta-Analyses extension for Scoping Reviews guidelines. Four databases (PubMed, CINAHL, PsycINFO, and Ichushi-Web) were searched for peer-reviewed studies published between 2010 and 2025. Studies focusing on the symptom experiences of adult patients with heart failure and their caregivers were included. Two reviewers independently screened and extracted the data, which were organised into main themes.

Of the 2,057 studies, 19 were included (14 qualitative, three cross-sectional, and two mixed-method). Findings were grouped into five categories: symptom experiences, in which breathlessness, tiredness, and swelling were often accepted as normal; detection and barriers, in which vague or small changes were often missed; family and caregiver roles, in which families sometimes encouraged early care-seeking but sometimes delayed it; management factors, including cognition, family support, healthcare complexity, and treatment burden; and knowledge and system gaps, including low body awareness, delays in severe cases, and limited attention to contextual factors.

Managing heart failure symptoms depends not only on medical knowledge but also on psychological, social, and contextual factors. Interventions should go beyond education by correcting normalisation, reducing the treatment burden, and combining human support with technology. Building multilayered strategies that link knowledge and action is essential for reducing readmissions and improving quality of life.

## Introduction and background

Heart failure is a chronic and progressive disease that affects millions of people worldwide and seriously impacts the lives of both patients and their caregivers [1]. Patients with heart failure often experience several symptoms simultaneously, such as breathlessness, fatigue, and swelling. These symptoms limit daily activities, cause psychological distress, and often lead to social isolation, resulting in a significant decrease in quality of life [2,3].

Therefore, promoting self-care behaviours among patients with heart failure is essential to prevent symptom worsening and improve clinical outcomes [4]. Among these behaviours, symptom perception, or the ability to notice and understand physical changes, is central to effective self-care management. Early recognition and appropriate response to symptoms can strongly influence health outcomes [5,6]. However, symptom perception is not only about medical knowledge; it depends deeply on how patients personally experience and interpret their symptoms.

Symptom experience is not limited to physical sensation. It includes aspects of self-perception, role performance, and social connections [3]. Fatigue is a continuous and disabling symptom that restricts daily life and decreases a patient's sense of self-efficacy. In addition, the appearance and management of symptoms are strongly associated with social determinants of health, such as access to health care, financial burden, and the presence of social support networks [7]. These findings show that the symptom experiences of patients with heart failure are complex processes involving biological, social, and emotional dimensions.

Despite this understanding, many studies have focused on symptom recognition or self-care behaviours, whereas few have systematically examined how patients live with and experience symptoms in their daily lives. Comprehensive research mapping the lived experiences of heart failure symptoms from the patient's perspective remains limited.

International clinical guidelines emphasise the importance of adopting a patient-centred approach to heart failure care [8]. Understanding patients' subjective experiences of symptoms is essential for designing effective medical interventions and building care systems that truly reflect the realities of patients' daily lives. A scoping review approach was selected to map the breadth of methodologically heterogeneous evidence on lived symptom experiences across qualitative, mixed-methods, and survey-based studies. Therefore, this scoping review aimed to explore and integrate existing studies on how patients with heart failure experience symptoms in their daily lives - encompassing both their subjective experiences and the perceptual processes through which they detect signs of worsening. Through this review, we aim to provide foundational knowledge for developing comprehensive and responsive nursing care models based on patients' lived experiences of symptoms.

To achieve this aim, three interrelated review questions were formulated, anchored in the lived experience of heart failure symptoms in daily life: (1) how adults with heart failure and their families experience and detect symptoms and signs of worsening in daily life; (2) what individual, social, and contextual factors shape how patients and their families manage these symptom experiences in everyday settings; and (3) what knowledge and system-level gaps hinder the translation of symptom experiences and detection into timely and appropriate responses.

## Review

Review questions

This scoping review addressed three interrelated questions anchored in the lived experience of heart failure symptoms in daily life: (1) How do adults with heart failure and their families experience and detect worsening symptoms in daily life? (2) What individual, social, and contextual factors shape how patients and their families manage these symptom experiences in everyday settings? (3) What knowledge and system-level gaps hinder the translation of symptom experiences and detection into timely and appropriate responses?

Methodology

Study Design

This scoping review followed the methodological framework proposed by Arksey and O’Malley [9], which was later refined by Levac et al. [10]. It was conducted in accordance with the Preferred Reporting Items for Systematic Reviews and Meta-Analyses extension for Scoping Reviews (PRISMA-ScR) guidelines [11].

This review aimed to systematically search, organise, and integrate existing studies that examined how patients with heart failure - across ejection fraction phenotypes (heart failure with reduced ejection fraction (HFrEF), heart failure with mildly reduced ejection fraction (HFmrEF), and heart failure with preserved ejection fraction (HFpEF)) - experience and manage their symptoms in their daily lives. To define the research questions and eligibility criteria, we applied the Population, Concept, and Context (PCC) framework as follows: Population - adult patients diagnosed with heart failure and their caregivers; Concept - symptom experiences in daily life; Context - adults living in community settings, excluding those receiving end-of-life or palliative care*.*

Eligibility Criteria

Studies were included if they met the following criteria: (1) targeted adult patients diagnosed with heart failure of any ejection fraction phenotype (HFrEF, HFmrEF, or HFpEF), or heart failure not otherwise specified, or their family members and caregivers; (2) explored symptom experiences in daily life; (3) were published as full-text, peer-reviewed articles in English or Japanese. The exclusion criteria were as follows: (1) studies that only examined the effects of symptom management interventions without exploring patients’ experiences; (2) studies involving patients living with mechanical circulatory support; (3) studies focusing on populations other than heart failure patients (e.g., caregivers or healthcare professionals only); (4) studies limited to end-of-life or palliative care settings; and (5) review articles (including integrative, narrative, systematic, and scoping reviews), to restrict the synthesis to primary evidence and avoid double-counting of findings already represented in included primary studies.

Search Strategy

A comprehensive literature search was conducted using four electronic databases: PubMed, CINAHL, PsycINFO, and Ichushi-Web. The search was performed from May 1 to May 31, 2025, and included studies published between January 1, 2010, and April 30, 2025. Eligible studies were limited to peer-reviewed articles published in English or Japanese, reflecting the linguistic competencies of the review team and thereby enabling reliable full-text assessment and nuanced interpretation of qualitative accounts. Search terms included combinations of keywords related to heart failure (e.g., “heart failure”), symptom experiences (e.g., “symptom experience,” “lived experience”), and daily life (e.g., “daily life,” “everyday life”). Boolean operators (AND, OR) were used to combine these terms. The complete PubMed search strategy is provided in Supplementary Appendix S1; equivalent strategies were adapted for the other databases. Additionally, reference lists of relevant studies were hand-searched to identify further eligible literature that may not have appeared in the database searches.​​​​​​

Selection of Sources of Evidence

The selection process was conducted in two stages. In the first stage, two independent reviewers (TF and JH) independently screened the titles and abstracts of all identified studies based on predefined eligibility criteria. Inter-rater reliability was assessed using both per cent agreement and Cohen’s κ; the observed agreement was 85.5%, and κ = 0.71 (95% CI: 0.68-0.75). In the second stage, the same reviewers independently assessed the full texts of the selected studies for eligibility. Disagreements were resolved through discussion, and when consensus could not be reached, a third reviewer (YM or YI) made the final decision. The selection process was illustrated using a PRISMA flow diagram.​​​​​​

Data Charting and Synthesis

Data from the included studies were collected using a standardised data extraction form developed by the research team. The form recorded key information such as author, year of publication, study design, country or region, participant characteristics, study objectives, symptom experiences explored, and major findings related to the review questions. Two reviewers independently extracted the data, and any discrepancies were resolved through discussion. Extracted data were synthesised using a descriptive qualitative approach: findings were summarised by key concepts, types of symptom experiences, and contextual factors, and were integrated both in tables and in descriptive text. The five thematic categories presented in the Results were derived inductively through iterative discussion among the reviewers. In this review, consistent with the primary aim of mapping the breadth rather than appraising the strength of available evidence, a formal critical appraisal of included studies was not conducted. To address the third review question, knowledge gaps were charted from the limitations and future research recommendations reported within the included studies.

Results

**Figure 1:**
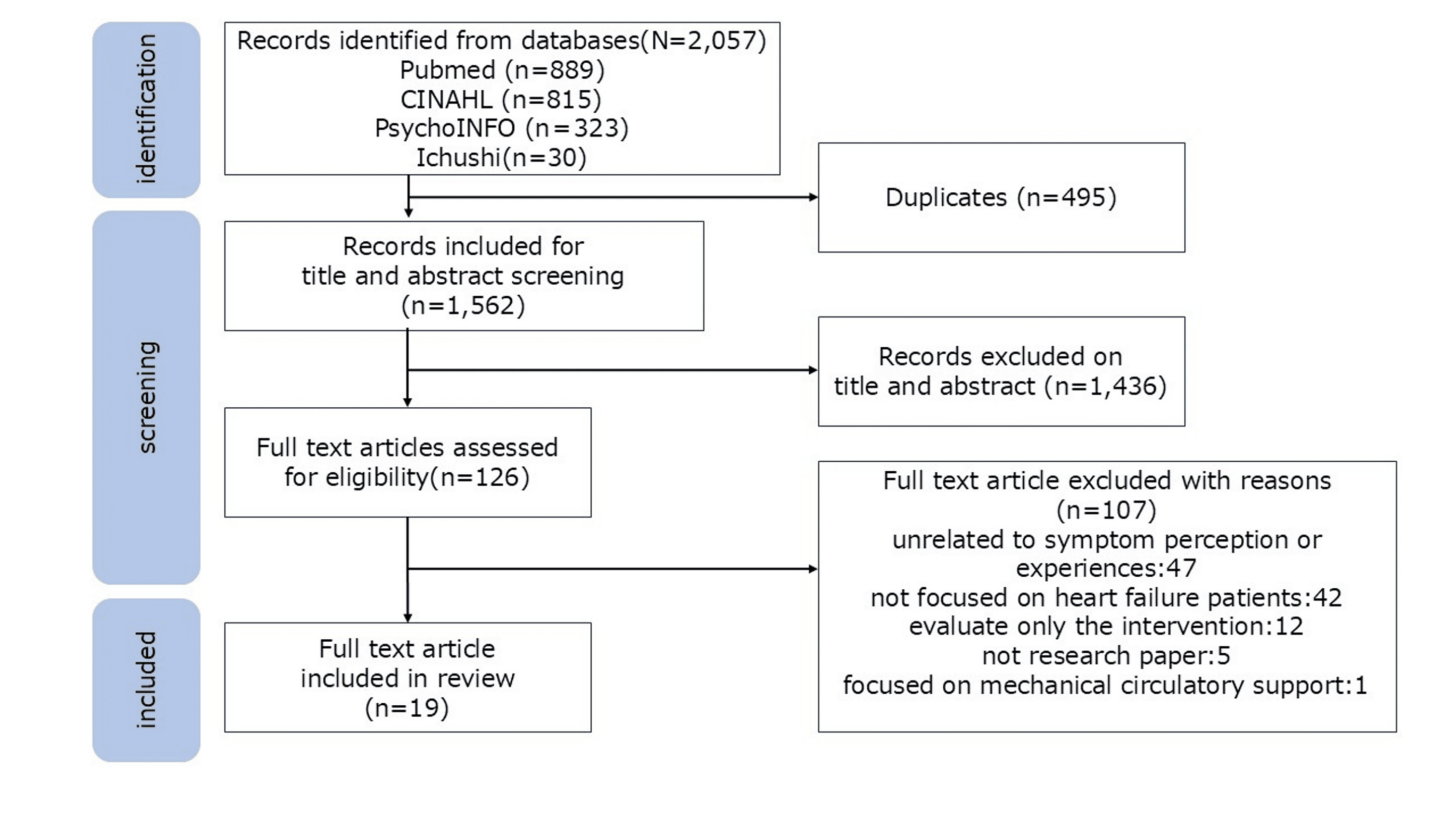
PRISMA-ScR flow diagram PRISMA-ScR: Preferred Reporting Items for Systematic Reviews and Meta-Analyses Extension for Scoping Reviews

A total of 2,057 records were identified in the four electronic databases. After removing duplicates, 1,562 articles were screened based on their title and abstract. Based on the inclusion and exclusion criteria, 1,436 studies were excluded. The remaining 126 full-text articles were reviewed in detail, and 19 studies were included in this scoping review (Figure 1). Of the 19 included studies, 14 were qualitative, three were cross-sectional, and two used mixed methods.​​​​​

The general characteristics of the included studies are presented in Table 1. Across the included studies, patients with heart failure and their families or caregivers described diverse and complex experiences related to symptoms and their recognition of worsening conditions. To provide an integrated understanding of these findings, the results were organised into five categories: (1) symptom experiences; (2) processes and barriers to symptom detection; (3) involvement of family and caregivers; (4) factors influencing symptom management; and (5) knowledge and system gaps.​​​​​

**Table 1:** Characteristics of included studies

Author, year, country	Design	Main focus	Key findings
Walthall et al. (2016) [12], UK	Qualitative	Heart failure patients' experience and management of breathlessness	Breathlessness is a central symptom of life for heart failure patients, involving fear and limitation, and patients cope with it alone while attempting to live with it.
Taylor et al. (2017) [13], UK	Qualitative	To explore symptom onset and diagnostic meaning of "heart failure" from the patient's perspective in the heart failure diagnostic pathway	Patients are prone to overlooking initial symptoms and experience intense anxiety regarding the diagnostic label "heart failure." Educational support promoting early recognition and understanding of the diagnosis is crucial.
Jones et al. (2012) [14], USA	Qualitative	The perceptions, experience, and meaning of fatigue as a distressing symptom of chronic heart failure.	Fatigue for heart failure patients is not merely tiredness but an internal experience embodying a "reduction of vital life-force" and "loss of physical strength." It is often poorly understood by others and accompanied by feelings of isolation.
Xu et al. (2018) [15], USA	Qualitative	Heart failure patients' symptom interpretation and response patterns.	Patients often fail to accurately judge the severity of their symptoms and tend to overlook ambiguous cues, especially because initial symptoms are frequently unrecognised.
Riegel et al. (2018) [16], USA	Mixed methods	Factors related to symptom recognition in chronic heart failure patients.	The ability of heart failure patients to recognise symptoms is influenced more by cognitive function, supportive environment, and decision-making skill than by knowledge alone. Support for symptom detection is key to preventing deterioration.
Palmer et al. (2023) [17], Australia	Qualitative	To explore in-depth experiences and emotions of people with heart failure within the context of self-care.	Fear, shame, and lack of understanding impede self-management, while heart failure patients live with "heartache" (emotional distress) and a "sense of failure”.
Shogaki et al. (2024) [18], Japan	Qualitative	Experience of acute exacerbation and recovery from heart failure.	Heart failure patients become intensely conscious of bodily abnormalities during acute exacerbation, and their central concern shifts to daily life during the recovery phase, but verbalising the experience deepens self-understanding.
Ivynian et al. (2024) [19], Australia	Mixed methods	How do illness perceptions influence coping responses and self-care behaviours that lead to delayed care-seeking?	Delayed care-seeking when heart failure patients experience symptoms is deeply related to inaccurate illness perceptions, such as attributing symptoms to "not being ill" or "age or fatigue," and emotion-focused coping strategies like denial and avoidance.
Schumacher et al. (2018) [20], Canada	Qualitative	Symptom recognition, interpretation, and response among heart failure patients living in the community.	Heart failure patients accept symptom worsening as "part of daily life" and cope by resting or adjusting their routine, consequently overlooking signs of deterioration. This process of symptom "normalization" is the background for delayed care-seeking and decreased functional capacity.
Riegel et al. (2013) [21], USA	Qualitative	Naturalistic decision making) and their course regarding symptoms in adults with chronic heart failure.	Decision-making in heart failure patients is an accumulation of "naturalistic decisions" rooted in experience, where self-management is formed through situation awareness, mental simulation of actions, and evaluation of outcomes.
Heo et al. (2021) [22], USA	Qualitative	Heart failure patients' symptom perception and support needs.	Patients responded to various symptoms like breathlessness and oedema using diverse strategies, including medication and position change, but many lacked knowledge, felt uncertain about efficacy, and often adopted a "wait-and-see" approach depending on the situation.
Lee et al. (2013) [23], USA	Cross sectional	The association between mild cognitive dysfunction and self-care/care-seeking behaviours in symptomatic heart failure patients.	Heart failure patients with mild cognitive dysfunction are less likely to translate perceived symptoms into action or care-seeking. Even mild cognitive impairment is a factor impeding self-management.
Min et al. (2023) [24], Korea	Qualitative	To explore in-depth experiences and emotions of people with heart failure within the context of self-care.	Heart failure patients engaged in "fragile yet continuous efforts" explored amidst anxiety from a sudden diagnosis and physical constraints, supported by family and healthcare providers. Psychological and family support served as motivation for self-care.
Sethares et al. (2014) [25], USA	Cross sectional	Factors associated with delay in seeking care for acute decompensated heart failure	Delay in seeking care among heart failure patients is influenced not by medical factors, but by psycho-social factors such as "wait-and-see" behaviour and the response of family members
Sano and Majima (2018) [26], Japan	Qualitative	Self-management process in elderly male patients with chronic heart failure who have avoided re-hospitalisation for over 2 years.	Elderly male heart failure patients strive to maintain their desired lifestyle while preventing symptom worsening, engaging in trial and error between personal preferences and constraints to achieve this.
Reeder et al. (2015) [27], USA	Qualitative	How heart failure patients perceive worsening symptoms and respond as self-care	Heart failure patients find it difficult to link vague bodily changes to their disease, often resorting to self-directed coping which can lead to deterioration.
Austin et al. (2022) [28], UK	Cross sectional	The association between heart failure symptoms and the burden of treatment (BoT)	The higher the severity of symptoms like breathlessness and fatigue in heart failure patients, the greater the perceived burden associated with self-care activities such as diet, exercise, and utilisation of healthcare services. Symptoms are not merely indicators of disease status, but significantly influence overall treatment and life, restricting patients' role function and quality of life.
Sevilla-Cazes et al. (2018) [29], USA	Qualitative	Patient and caregiver challenges to heart failure home management and perceived reasons for readmission	Instead of "failing to follow instructions," patients are "attempting to come to terms with the illness in their own way." Readmission is not a "self-management failure" but a rational choice where the patient assesses their limits and seeks to regain safety.
Hall et al. (2014) [30], USA	Qualitative	Heart failure patients’perceptions and use of technology to manage disease symptoms.	While heart failure patients are accustomed to in-home measuring devices, there are psychological and practical barriers to using information and communication technology (ICT) or remote monitoring. Promoting technology utilisation requires the prerequisites of ensuring patient trust, understanding, and a supportive environment.

Symptom Experiences

Across the included studies, patients with heart failure described symptom experiences that were frequent, burdensome, and deeply embedded in daily life. Breathlessness and fatigue were among the most commonly reported symptoms. Shortness of breath (85%), fatigue (53%), and oedema (42%) were the most frequently recognised pre-admission symptoms among the 60 patients interviewed by Reeder et al. [27], and breathlessness on exertion affected 82% and fatigue 85% of the 333 community-dwelling patients in the SYMPACT survey [28]. However, the meaning patients attributed to these symptoms was often more important than the symptoms themselves. Symptom experiences were not simply physical events; rather, they involved subjective interpretation, emotional responses, and ongoing adjustments to everyday living.

Breathlessness was the most pervasive and disabling symptom, reported as a daily experience across virtually all included studies [12]. Its character differed markedly across disease phases: acute episodes were described in terms of terror, suffocation, and a sense of drowning, whereas chronic breathlessness gradually constricted the texture of everyday life, limiting activities such as bathing, climbing stairs, and sleeping. Night-time was particularly burdensome, with patients lying awake in fear, unable to lie flat. Despite its severity, many patients under-reported breathlessness to healthcare professionals and concealed its extent from family members, often managing it through self-devised strategies such as adjusting posture or slowing their pace [12]. Patients tended to seek help only when breathlessness became acute or severely disrupted daily activities; gradual increases in breathlessness during routine exertion were frequently attributed to ageing, deconditioning, or comorbid conditions rather than cardiac deterioration [13].

Fatigue was similarly prevalent but proved far more resistant to accurate interpretation. Rather than using clinical terminology, patients preferred expressions conveying low energy, physical depletion, or a loss of vital force [14]. Because fatigue was experienced as an entirely internal phenomenon - invisible to family, friends, and clinicians alike - patients frequently felt misunderstood, isolated, and reluctant to raise the symptom in healthcare encounters. The existential dimension of fatigue was particularly striking: it functioned as an embodied barometer of mortality, experienced as a diminishment of life itself rather than a symptom to be reported and treated [14]. Across multiple studies, fatigue was consistently misattributed to ageing or overexertion, a pattern that significantly delayed recognition of clinical deterioration [13,14].

While patients were monitored for fluid retention through daily weighing and ankle inspection, these objective indicators were unreliable guides to action. When presented with vignette scenarios, patients could identify acute breathlessness as requiring urgent intervention but struggled considerably with more gradual symptoms such as weight gain and ankle swelling, often responding with palliative measures rather than seeking clinical advice [15]. Consistent with this, longitudinal monitoring revealed that nearly half of patients showed a mismatch between objectively measured thoracic fluid accumulation and their own subjective perception of congestion [16]. Those who interpreted symptoms more accurately engaged in what has been termed body listening - an attentive, experience-based attunement to subtle internal signals - and demonstrated higher decision-making skill and stronger social support [16].

The emotional dimensions of symptom experience were equally consequential. Living with heart failure generated a sustained burden of fear, uncertainty, shame, and guilt that shaped daily decision-making and healthcare engagement [17]. Both the experience of symptoms and the diagnostic label “heart failure” itself evoked associations with imminent death, in some cases discouraging patients from discussing their condition openly with family or clinicians [13,17]. In a phenomenological study conducted in Japan, patients reflecting on the period preceding emergency hospitalisation recalled vague bodily signals - unusual fatigue, changes in urine character, hoarseness - that had remained unrecognised as signs of worsening, illustrating how early warning symptoms are readily absorbed into the background of everyday life rather than interpreted as clinically meaningful [18].

Processes and Barriers to Symptom Detection

Patients consistently experienced difficulty in recognising the worsening of heart failure symptoms, and this difficulty operated through several interrelated mechanisms rather than a single barrier.

A primary and cross-cutting barrier was the progressive normalisation of symptoms through daily habituation. Symptoms initially perceived as alarming became incorporated into the fabric of everyday life as they recurred without acute consequence [19,20]. Patients built tolerance incrementally - comparing new sensations against a continuously adjusted baseline of familiarity - while simultaneously enacting three active coping strategies through which normalisation was reinforced: resting to resolve symptoms in real time, reorganising daily routines to avoid symptomatic episodes, and progressively relinquishing valued roles [20]. Although each strategy temporarily restored a sense of control, their cumulative effect was a reduction in exertional activity that obscured the physiological signals necessary for accurate self-monitoring.

Closely linked to normalisation was the systematic misattribution of symptoms to non-cardiac causes. Breathlessness, fatigue, and oedema were consistently attributed to ageing, deconditioning, or comorbid conditions rather than cardiac deterioration [19,20]. This misattribution was not simply a knowledge deficit; it was reinforced by ambiguous symptom presentations, particularly when overlapping conditions such as respiratory illness or constipation produced sensations indistinguishable from heart failure exacerbation [19]. In the absence of clear perceptual anchors, patients defaulted to wait-and-see behaviour - monitoring symptoms passively while deferring decisions according to severity, mood, or the availability of family members - a pattern documented consistently across qualitative and mixed-methods studies [20-22].

Cognitive function emerged as a critical modifying variable in this process. Using a cardiovascular-specific cut-off on the Montreal Cognitive Assessment, patients with mild cognitive dysfunction demonstrated 21.5% worse self-care management scores and 51% worse consulting behaviours compared with cognitively intact counterparts [23]. The brain regions most affected by the haemodynamic consequences of heart failure overlap substantially with those governing decision-making, suggesting that cognitive vulnerability operates as a largely invisible amplifier of the barriers described above.

Underpinning all of these processes was the naturalistic character of self-care decision-making itself. Rather than reasoning analytically about symptoms, patients relied on situation awareness and mental simulation grounded in personal experience [21]. Crucially, uncertainty and ambiguity - both pervasive features of heart failure symptom presentation - consistently directed this process toward passive responses. Comparison with previous hospitalisations reinforced inaction: patients who judged their current condition as less severe than a prior crisis concluded that intervention was premature [21].

The included studies also described circumstances in which detection proceeded more successfully. Patients who interpreted and responded appropriately to worsening symptoms were distinguished by higher decision-making skill and better-quality social support, in contrast to those who struggled with recognition and action [16]. The post-recovery period following an acute exacerbation emerged as a distinctive window in which patients could articulate and contextualise prior bodily experiences when prompted, even though these same experiences had remained below the threshold of conscious awareness during routine daily life [18]. Accumulated experience with prior symptomatic episodes further supported detection: memory of concrete physical sensations, emotions, and imagery allowed some patients to rapidly re-appraise new symptoms, shortening appraisal and illness delays even when initial attributions had been inaccurate [19].

These findings collectively indicate that symptom detection in heart failure is neither linear nor primarily knowledge-dependent. It is shaped by the erosion of perceptual sensitivity through normalisation, cognitive vulnerability, and the inherent uncertainty of experiential decision-making in daily life - a combination that frequently delays recognition until deterioration becomes critical.

Involvement of Family and Caregivers

Family members and caregivers played a structurally ambivalent role in heart failure symptom management - simultaneously enabling and impeding timely care-seeking - and this duality was a consistent finding across both qualitative and quantitative studies.

The facilitative dimension of family involvement was most evident in crisis situations. When symptoms reached a threshold that could no longer be managed at home, families actively intervened: accompanying patients to consultations, contacting emergency services, and taking immediate action to support patients [24,25]. Family members were consulted in 87% of cases when patients first noticed symptom changes, and an active family response was associated with significantly shorter delay times compared with a passive one [25]. Caregivers also monitored patients' daily activities, helped manage dietary restrictions, and functioned as a practical scaffold for self-care - particularly for older adults for whom independent management had become physically challenging [24].

However, family involvement also generated its own barriers. When mild or gradual symptoms were present, both patients and those around them tended toward wait-and-see responses, and a passive response from others was identified as an independent predictor of treatment-seeking delay, increasing delay time by nearly 38 hours compared with an active response [25]. Patients' pervasive shame, guilt, and fear of being a burden led them to actively conceal symptoms from family members, particularly those who had witnessed prior cardiac events and responded with heightened anxiety [17]. Paradoxically, family members' visible worry and protectiveness sometimes contributed to patients' emotional distress and could reinforce concealment rather than disclosure [17]. This relational complexity was also captured in the dynamic of conflict and gratitude, whereby, despite genuine appreciation for caregivers' support, sustained dependency generated interpersonal friction and diminished patients' sense of autonomy [24].

Locally embedded social norms also influenced symptom management within everyday relationships. In a qualitative study of older male patients in Japan, self-management was shaped by social obligations that frequently competed with medical recommendations [26]. Adherence to dietary and lifestyle advice was negotiated against contextual demands such as workplace meals, alcohol consumption at social gatherings, and the maintenance of established role identities. Patients tended to adopt a process of trial and error, with selective disclosure of their condition and partial dietary modification rather than strict adherence [26]. These observations indicate that locally specific social norms may operate as additional determinants of self-management alongside family support and caregiver behaviour.

Taken together, these findings indicate that the contribution of family and social context to symptom management in heart failure is neither uniformly supportive nor uniformly obstructive. Its character depends on the emotional climate between patients and caregivers, the severity and visibility of symptoms, and the social and contextual norms that frame what constitutes acceptable help-seeking and disclosure.

Factors Influencing Symptom Management

Symptom management in daily life was shaped by an interplay of individual, social, structural, and technological factors, none of which operated in isolation.

At the individual level, cognitive function was a central determinant of whether perceived symptoms were translated into self-care action. Patients with even mild cognitive impairment demonstrated blunted responses to worsening symptoms and were substantially less likely to initiate care-seeking behaviours compared with cognitively intact counterparts [23]. Illness perception operated alongside cognition as a distinct barrier: patients who attributed symptoms to ageing, fatigue, or temporary states - rather than to heart failure - tended to adopt emotion-focused coping strategies such as denial and avoidance, which structurally delayed care-seeking [19]. Crucially, even when patients sensed that something was not quite right, many were unable to specify what was wrong, and instead attributed their deteriorating condition to changes in diet or medications rather than to worsening disease [27]. This misattribution meant that more than 40% of patients experienced symptoms for at least two weeks before seeking care [27]. Decision-making in this context was rarely analytical; rather, it followed a naturalistic pattern grounded in situation awareness and mental simulation derived from accumulated personal experience [21].

Social factors functioned as both protective and inhibitory forces. When family members drew attention to symptom changes, patients were more likely to seek care early; conversely, families responding passively - whether adopting a wait-and-see approach or not encouraging medical contact - substantially delayed help-seeking [25]. Beyond crisis response, the ongoing presence of family caregivers served as an essential source of practical and emotional support, providing patients with the motivation to sustain daily self-care efforts even amid physical constraints and uncertainty [24]. For patients living alone, this protective function was largely absent, and competing domestic obligations - such as caring for pets or dependants - could directly compete with care-seeking, leading to further delay [19].

Structural and system-level factors compounded individual vulnerabilities. Complex medical access routes, insufficient discharge education, and confusing self-management protocols collectively increased the burden of treatment, eroding patients' capacity for home-based self-care [28,29]. Quantitative evidence from the SYMPACT study demonstrated this burden concretely: among the specific domains of self-care engagement, exercise carried the highest burden score, followed by diet and difficulty with healthcare services - domains in which over half of patients reported receiving no professional guidance [28]. Furthermore, the emotional impact of engaging with self-care regimens showed a moderate-to-strong association with symptom severity, suggesting that the psychological cost of maintaining daily management routines may itself become a barrier to adherence [28]. Importantly, readmission in this context was not primarily a failure of adherence; patients often engaged in a cycle of limit testing, in which uncertain symptom improvement and unclear instructions led to progressive reduction in self-management capacity, and hospitalisation ultimately became a rational choice made from a position of distress rather than negligence [29]. A parallel cycle of despair was also identified: patients who experienced worsening symptoms despite perceived good adherence developed hopelessness and frustration, which further eroded their self-management behaviours [29]. Non-pharmacological self-care, including salt and fluid restriction, daily weighing, and exercise, was similarly constrained by the interacting patient-, condition-, therapy-, and system-level factors captured in the treatment-burden framework [29].

Technological tools emerged as a potential resource for bridging these gaps; however, their integration into daily symptom management remained uneven. Although patients were generally familiar with and used over-the-counter home monitoring devices such as blood pressure cuffs and scales, daily weighing - a cornerstone of heart failure self-management - remained poorly maintained, in part because patients failed to associate it with fluid retention and disease worsening [30]. Barriers to broader technology use included financial costs, mistrust of device-generated information, low digital literacy, privacy concerns, and a strong preference for physician-validated information over independent use of digital tools [30]. Notably, technology acceptance was considerably higher when its use was initiated or endorsed by healthcare providers, suggesting that technology integration must be embedded within relational care rather than offered as a standalone solution [30]. These findings collectively indicate that effective symptom management cannot be achieved through any single strategy but requires multilayered support that simultaneously addresses personal interpretation, relational context, system complexity, and the conditions under which patients can meaningfully and safely engage with technology. 

Knowledge and System Gaps

The studies included in this review collectively reveal persistent gaps at both the individual knowledge level and the healthcare system level, which together sustain a knowledge-action gap - the distance between what patients nominally understand about heart failure and what they can act upon in daily life.

At the level of somatic knowledge, a fundamental problem is the disconnect between explicit disease understanding and embodied symptom awareness. Riegel et al. demonstrated this gap objectively: 44% of patients showed a symptom-hemodynamic mismatch, with objective evidence of fluid retention failing to correspond to subjective symptom perception [16]. Qualitative analysis from the same study identified two distinct response patterns - patients able to interpret and respond appropriately to symptoms were characterised by higher decision-making skill and better quality social support, whereas those who struggled tended to attribute symptoms to comorbidities, await the emergence of expected patterns, and delay treatment for days or more [16]. Underlying this objective-subjective gap is a deficit in somatic awareness itself. Patients often reported a vague sense that “something was not quite right” but were unable to specify the underlying change and commonly misattributed symptoms to diet or medication changes rather than to worsening heart failure, with over 40% experiencing symptoms for at least two weeks before seeking care [27]. The translation of such awareness into action was further blunted in patients with mild cognitive dysfunction, who showed 21.5% worse self-care management and 51% worse consulting behaviours than their cognitively intact counterparts [23].

Shogaki et al.'s phenomenological study extended this insight by revealing that patients could recall pre-hospitalisation bodily changes - including altered urine characteristics, subtle throat discomfort, and diffuse malaise - only when explicitly prompted during the post-recovery period [18]. In daily life, these experiences had remained below the threshold of conscious awareness, absorbed within habitual bodily routines. This finding suggests that somatic knowledge does not develop automatically through disease experience; without active facilitation, the body's early warning signals remain unarticulated and therefore unactionable [18].

Compounding this, post-discharge education practices were insufficiently targeted to support patients' ability to bridge this gap. Prior episodes of acute exacerbation did not reliably translate into improved symptom perception in subsequent episodes; patients tended to re-normalise recurrent symptoms rather than update their interpretive framework [19,20]. Riegel et al. further identified that patients with poor self-care maintenance were not necessarily symptomatic, suggesting that standard educational approaches focused on symptom checklists may fail to develop the body listening skills that characterise more capable self-managers [16]. Shogaki et al. similarly showed that the period immediately following recovery from acute exacerbation represents a unique window for education, during which patients - when asked - could begin to articulate and contextualise their bodily experiences, potentially establishing a foundation for improved symptom perception after discharge [18]. Xu et al. confirmed that deficits in somatic awareness were not consistently addressed by routine heart failure education [15].

At the system level, structural barriers further widened the knowledge-action gap. Healthcare complexity - including competing demands from multiple comorbidities, fragmented follow-up arrangements, and unclear guidance on when to contact providers - contributed substantially to delayed care-seeking even among patients who had recognised symptom changes [29]. These system-level factors interacted with individual knowledge deficits in mutually reinforcing ways: patients uncertain whether their symptoms warranted action were simultaneously navigating a system that provided insufficient behavioural guidance. Beyond these structural factors, the contextual fit of self-care guidance shaped what patients could realistically enact. Sano and Majima illustrated this vividly among older Japanese men with heart failure, who continually negotiated between medical recommendations and locally embedded social obligations. Avoiding meals out with colleagues, drinking at workplace gatherings, and restricting traditional high-sodium foods such as sashimi with soy sauce or pickled dishes proved deeply difficult. Many consequently prioritised social belonging and personal preferences over strict adherence, accepting some risk of decompensation as an acceptable trade-off [26]. Such contextually mediated trade-offs illustrate how self-care guidance developed without attention to local dietary practices, social roles, and gendered expectations may be only partially actionable, narrowing its applicability in heterogeneous populations.

Taken together, these findings indicate that bridging the knowledge-action gap in heart failure requires more than information transfer. Education must be experientially grounded - supporting patients in articulating and connecting their own bodily experiences to their disease - and delivered at strategically optimal moments, particularly during the post-recovery period, when patients are most receptive to reflecting on and reinterpreting their bodily experience [18]. At the system level, contextually responsive guidance and clear behavioural protocols for symptom response are essential if improved awareness is to translate into timely action.

Discussion

This scoping review integrated the findings of 19 studies that explored how patients with heart failure and their families or caregivers experience, detect, and manage symptoms in daily life, and the knowledge gaps that influence these processes. This review revealed that symptom experiences in heart failure are not merely medical or biological events. They are deeply shaped by subjective perceptions, family dynamics, social backgrounds, and healthcare system constraints, such as complex access routes and insufficient discharge education. These intertwined factors demonstrate that symptom management in heart failure is a multidimensional process that goes beyond clinical explanations. The following discussion addresses three perspectives based on the review questions: (1) symptom experiences and detection of worsening signs; (2) individual, social, and contextual factors influencing symptom management; and (3) knowledge and system gaps related to symptom experiences, detection, and responses.

Symptom Experiences and Detection of Worsening Signs

Symptom experiences in patients with heart failure were closely associated with daily restrictions and psychological distress, in addition to typical symptoms such as breathlessness, fatigue, oedema, and sleep disturbance. McHorney et al. [1] reported that these symptoms often interfered with social activities and family interactions and were associated with anxiety and depression. Patients tended to accept such changes as a new normal; however, this acceptance sometimes increased the risk of overlooking worsening signs or delaying medical visits. Fry et al. [31] reported that patients frequently confused early symptoms with other illnesses or ageing, resulting in a delayed response. For patients with multiple comorbidities, it was difficult to determine whether cough or fatigue indicated heart failure deterioration.

Thus, recognising symptoms is not simply sensory awareness but a process that involves cognitive interpretation and adaptation to daily life. Santos et al. [5] defined symptom detection as a complex process of monitoring bodily changes, interpreting their meaning, and linking them to actions, noting that many patients struggle with this process. This review confirmed that symptom recognition is shaped by personal experience and life context rather than physical sensations alone. What patients consider abnormal differs individually and is strongly influenced by their daily routines and past experiences. Therefore, effective support for early detection should respect how patients give meaning to their experiences while helping them transform awareness into concrete self-care behaviours.

While much of the included literature foregrounded barriers to detection, our synthesis also identified experiential learning from prior episodes, decision-making skill coupled with responsive social support, and the post-recovery period as recurrent mechanisms underlying successful detection [16,18,19]. These positive mechanisms suggest that strategies to improve timely symptom response should not focus solely on removing barriers but also on actively strengthening the experiential, relational, and reflective resources through which detection succeeds. Importantly, the included studies did not report formal metrics of detection accuracy, timeliness, or sensitivity; characterising successful detection through such measures remains an important direction for future primary research.

Individual, Social, and Contextual Factors Influencing Symptom Management

Symptom management in patients with heart failure is influenced by individual psychological traits and living conditions, as well as by social resources, contextual factors, and patients' experience of healthcare complexity. Clark et al. [32] demonstrated that successful self-management, such as weight monitoring and salt restriction, was dependent not only on knowledge or motivation but also on family support and relationships with healthcare providers. Thus, self-care is not performed by the patient alone; rather, the availability of daily support strongly affects whether these behaviours can be maintained.

For patients with multiple chronic conditions, complex medication schedules and the need to visit several clinics are often significant barriers [33]. Such burdens can reduce motivation for self-care and increase the risk of worsening symptoms. Symptom management is often perceived as an overly complicated task by older adults and those with limited social resources. Greenhalgh et al. [34] noted that although technologies such as remote monitoring could make symptom management more efficient, some patients perceive these systems as cold care, which undermines a sense of reassurance. This suggests that the introduction of technology does not always align with patients' personal values or expectations of care.

Health-seeking behaviours and self-management were influenced by sex, age, and social background; however, these factors showed inconsistent patterns across studies [35]. Overall, heart failure management is shaped by the interplay between systemic constraints and social contexts, requiring multilayered approaches beyond individual-level interventions.

Knowledge and System Gaps Related to Symptom Experiences, Detection, and Responses

The included studies suggest several recurring gaps in the clinical, cognitive, and contextual understanding of how patients with heart failure experience, detect, and respond to symptoms. These gaps were derived through interpretive synthesis of the included studies, consistent with the scoping review framework; systematic mapping against an established self-care theory would be a valuable direction for future research. Self-management of heart failure involves multiple processes - monitoring, recognition, interpretation, and response - which are inherently difficult for many patients. Patients with low somatic awareness may delay seeking medical attention when their symptoms worsen, which increases the risk of cardiovascular events. When patients lack sufficient knowledge, they may misinterpret breathlessness or fatigue as a result of stress or ageing, hindering appropriate self-care behaviours [5].

Patients with New York Heart Association (NYHA) Class III or IV heart failure tend to become accustomed to their symptoms, leading to a slower recognition of deterioration and delayed hospital visits [36]. This finding highlights the need for further studies to clarify the mechanisms underlying the delay in patients with advanced disease. Moreover, Schumacher et al. [20] reported that even after receiving education, patients continued to normalise their symptoms, indicating that knowledge alone was insufficient for behavioural change. This suggests the presence of cognitive barriers between symptom recognition and action, underscoring the need to improve educational approaches.

Patients' contextual beliefs and social values shape how they understand and respond to symptoms; however, the included studies suggest that these factors have received limited attention in existing heart failure research [37]. Future strategies should focus on understanding patients' contextual and social perspectives and incorporating them into care. Multilayered interventions that combine contextually responsive education and personalised support may be needed to bridge the gap between cognitive understanding and concrete behavioural change.

Limitations

This review has several methodological limitations. First, a formal critical appraisal of included studies was not conducted, in keeping with the aim of mapping the breadth of available evidence; consequently, the synthesis should be read as a descriptive map rather than as a comparative assessment of study quality. Second, inclusion was not stratified by ejection fraction phenotype, as most primary studies did not report phenotype-specific symptom data, and our findings therefore reflect heart failure as a broad clinical entity. Third, eligibility was restricted to peer-reviewed articles in English and Japanese, reflecting the review team's linguistic competencies; evidence from regions whose dominant languages fall outside this scope may be underrepresented, limiting the geographic and cultural diversity of the synthesis.

## Conclusions

This scoping review revealed that the processes of experiencing, detecting, and responding to symptoms among patients with heart failure are not merely biological phenomena. They are closely shaped by patients' subjective interpretations, family relationships, and perceptions of healthcare complexity, with social and contextual factors also likely to play a role. Patients often perceive vague and gradually progressing symptoms as part of their daily life. Whether patients notice these signs is shaped not only by medical knowledge but also by the personal meanings and rhythms of everyday living. Symptom management is influenced not only by individual factors such as cognitive function and self-efficacy, but also by social and healthcare-related factors, including family involvement, treatment burden, complexity of care pathways, and attitudes toward technology. Low somatic awareness, delayed care-seeking in advanced cases, and limited attention to social and contextual factors may further contribute to a gap between knowledge and action.

For clinical practice, several practical implications emerge from these findings: educational programs that help counter normalisation bias through family participation or scenario-based learning; consistent post-discharge action plans and clear communication systems; reduction of treatment burden through simplified medication and visit schedules; use of technology in ways that preserve human relationships rather than creating cold care; and individualised support that considers patients' personal and social contexts. Future research should longitudinally examine how patients' meaning-making leads to care-seeking behaviour within family, social, and system contexts, and evaluate multilayered interventions that bridge the gap between recognition and action in symptom management.

## Appendices

Appendix S1

PubMed Search Strategy

Database: PubMed Search date: May 2025 Publication date filter: 1 January 2010 to 30 April 2025 (applied via PubMed's date limit) Language filter: English

Search string: ("heart failure"[MeSH] OR "heart failure"[tiab] OR "cardiac failure"[tiab]) AND ("symptom experience"[tiab] OR "lived experience"[tiab] OR "symptom perception"[tiab] OR "symptom recognition"[tiab] OR "symptom management"[tiab] OR "illness experience"[tiab] OR "patient experience"[tiab] OR "subjective experience"[tiab] OR "symptoms"[tiab]) AND ("daily life"[tiab] OR "everyday life"[tiab] OR "activities of daily living"[MeSH] OR "self care"[MeSH] OR "self-management"[tiab] OR "quality of life"[MeSH] OR "patient perspective"[tiab] OR "patient-centered"[tiab]) AND (English[lang]) NOT ("palliative care"[MeSH] OR "terminal care"[MeSH] OR "hospice care"[MeSH] OR "end of life"[tiab])

Notes: [MeSH] = Medical Subject Heading (controlled vocabulary); [tiab] = title/abstract field tag; [lang] = language filter

Japanese-language literature was retrieved separately via Ichushi-Web using equivalent concept blocks adapted for that database's syntax. Equivalent search strategies were adapted for CINAHL and PsycINFO according to each database’s syntax and indexing system.
